# Primary squamous cell carcinoma of the endometrium associated with human papilloma virus in a young woman: a case report

**DOI:** 10.1186/s13256-019-2098-4

**Published:** 2019-06-01

**Authors:** Tchin Darré, Abdoul-Samadou Aboubakari, Lantam Sonhaye, Baguilane Douaguibe, Akila Bassowa, Gado Napo-Koura

**Affiliations:** 1 Department of Pathology, University Teaching Hospital of Lomé, Lomé, Togo; 2 Department Obstetrics and Gynecology, University Teaching Hospital of Lomé, Lomé, Togo; 3 Department of Radiology, University Teaching Hospital of Lomé, Lomé, Togo; 4 University of Lomé, BP 1515, Lomé, Togo

**Keywords:** Endometrial biopsy, Squamous cell carcinoma, Human papillomavirus, Immunohistochemical staining, Africa

## Abstract

**Background:**

Primary squamous cell carcinoma of the endometrium is an extremely rare tumor with poorly understood pathogenesis.

**Case presentation:**

We report a case of a 28-year-old Togolese woman who had consulted for vaginal bleeding and pelvic pain. Ultrasound showed thickening of the lining of the endometrium, and biopsy curettage was done. Anatomopathological examination was noteworthy for a proliferation of squamous cells often connected by union bridges arranged in tumor lobules with dyskeratotic maturation. Immunohistochemistry showed epithelial membrane antigen positivity, anti-pancytokeratin 1 markers of tumor cells positivity, chromogranin A negativity, actin negativity, S100 negativity, estrogen receptor negativity, and progesterone receptor negativity. *In situ* hybridization had objectified human papillomavirus genotypes 16/18. The diagnosis of primary squamous cell carcinoma of the endometrium associated with human papilloma virus was retained. A hysterectomy was performed, and the tumor was classified pT1N0M0.

**Conclusion:**

The pathogenesis of this endometrial cancer is complex, and its association with human papillomavirus does not explain its genesis.

## Background

Primary squamous cell carcinoma (PSCC) of the endometrium is an extremely rare tumor [1]. Its prevalence is approximately 0.1% [2]. The condition has been strongly associated with pyometra, cervical stenosis, multiparity, and chronic inflammation [2, 3]. Its genesis, histogenesis, and biological behavior are unknown [3]. The diagnostic criteria for PSCC of the endometrium include the absence of the following: (1) coexisting endometrial adenocarcinoma; (2) a connection between squamous cell carcinoma (SCC) of the endometrium and the squamous epithelium of the cervix; and (3) a primary squamous lesion in the cervix, either *in situ* SCC or an invasive carcinoma [3, 4]. Human papillomavirus (HPV) infection has been associated with the development of cervical epidermoid carcinoma, but its involvement in endometrial cancers is poorly documented [2, 4]. Given the extreme rarity of PSCC of the endometrium and the fact that all the previously reported cases have been in menopausal women, we report a case of exceptional localization of PSCC of the endometrium associated with HPV in a young Togolese woman.

## Case presentation

A 28-year-old Togolese woman was admitted to the Gynecology Department of Kara University Hospital with a 1-week history of bleeding and pelvic pain. She reported her age at first menstrual period as 14 years old and her age at first sexual intercourse as 15 years old. She did not smoke, drink alcohol, or take contraceptive pills. She did not report any history of sexually transmitted infections. The patient had no medical, obstetric, social, environmental, or special family history. She had never received an intervention, and she had a good psychosocial state.

Clinical examination revealed an axillary temperature of 38 °C, body weight of 54 kg, and height of 1.68 m. The patient’s blood pressure was 100/60 mmHg, and her pulse was good. Her general condition was good. Upon inspection, her conjunctivas were moderately hyperemic, and her abdomen palpated normally but was painful on palpation of the pelvic region. There was palpable lymphadenopathy in the region of the inguinal lymph nodes. Her gynecological speculum examination showed a macroscopically healthy uterine cervix. The result of examination of her external genitalia was normal. Her neurological examination and other investigations were unremarkable. The result of biological explorations, namely hemoglobin, was normal (13.5 g/dl). Her renal biology was normal (urea 0.22 g/L, creatinine 9 mg/L, blood glucose 0.9 g/L). The result of her hepatic evaluation was also normal (transaminases 19 IU/L, phosphatases 104 IU/L, γ-glutamyltransferase 21 IU/L). The result of her human immunodeficiency virus serology test was negative.

**Fig. 1:**
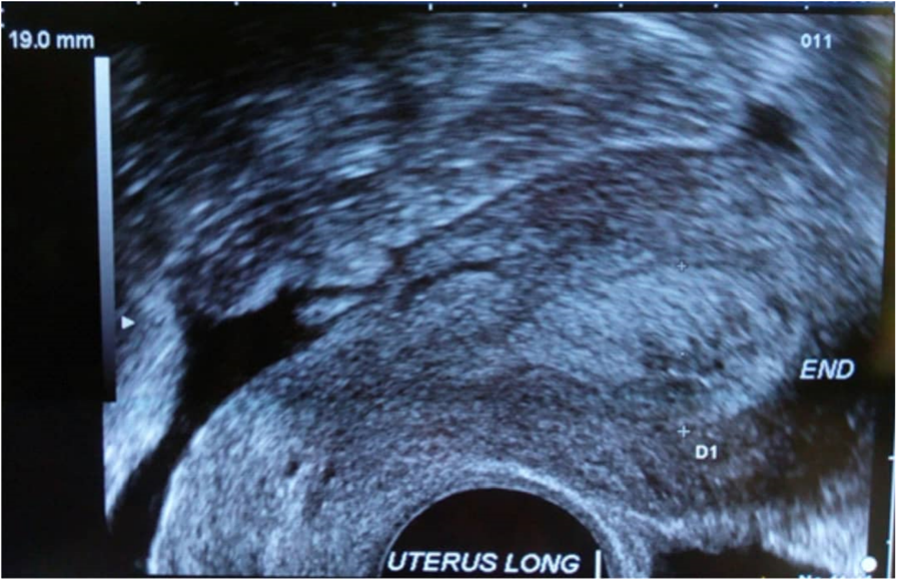
Endovaginal ultrasound image of a longitudinal section of the uterus showing thickening of the endometrium measuring 19 mm

Abdominal ultrasound showed regular thickening of the endometrium, measuring 19 mm thick, and no substantial masses (Fig. 1). Her ovaries were normal in size. Biopsy with curettage of the endometrium was performed.

**Fig. 2:**
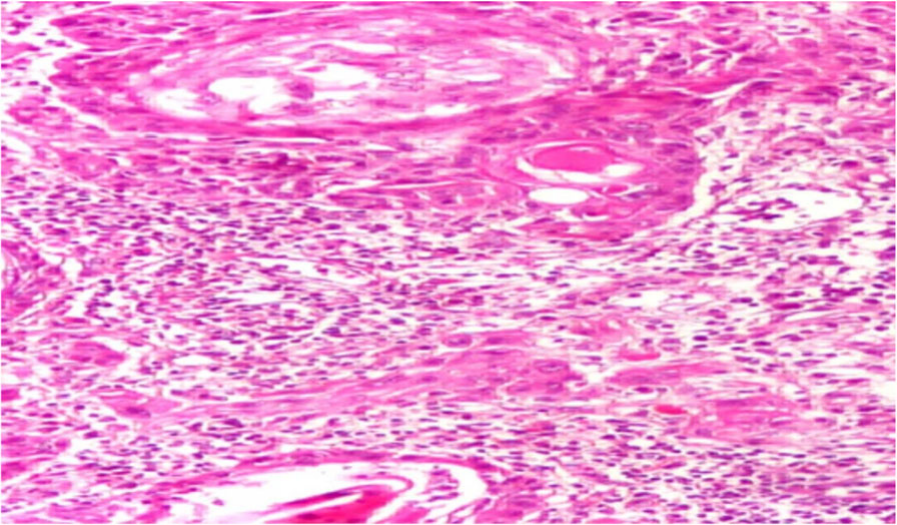
Differential squamous cell carcinoma of the endometrium. Note the centric tumor lobes of dsykeratotic maturation (hematoxylin and eosin stain, original magnification ×100)

**Fig. 3:**
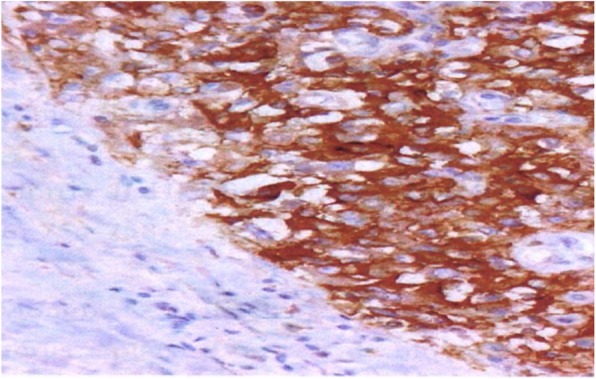
Diffuse positivity for anti-epithelial membrane antigen tumor cells as seen by immunohistochemistry (original magnification ×100)

**Fig. 4:**
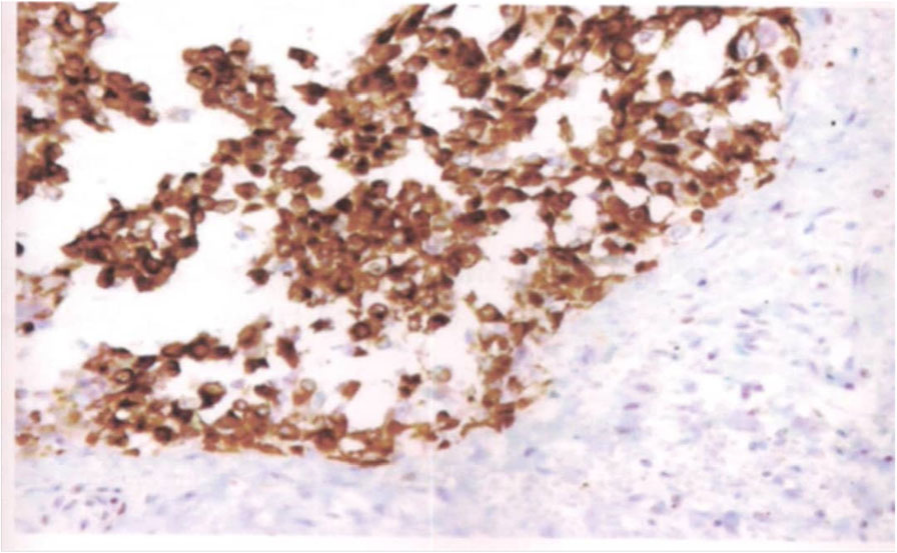
Focal positivity for anti-pancytokeratin 1 marker of tumor cells seen by immunohistochemistry (anticytokeratin stain, original magnification ×100)

Anatomopathological examination with hematoxylin and eosin (H&E) staining showed neoplastic cells of an epidermoid nature with bridges of unions arranged in lobules, often centered, with dyskeratotic maturation. Cellular atypia of epidermoid cells, particularly anisokaryosis, and mitotic figures were noted (Fig. 2). Immunohistochemistry (IHC) investigations showed positivity for anti-epithelial membrane antigen markers and anti-pancytokeratin 1 (KL1+) markers of tumor cells (Figs. 3 and 4). The cells were negative for chromogranin A, actin, S100, estrogen receptor (ER), and progesterone receptor (PR). Positive staining for Ki-67 (antigen of cell multiplication) was observed in 50% of the tumor cells. *In situ* hybridization demonstrated HPV genotype 16/18. A diagnosis of PSCC of the endometrium associated with HPV genotype 16/18 was made.

Total hysterectomy with bilateral adnexectomy and bilateral inguinal dissection were performed. The inguinal dissection revealed a total of five ganglia (three lymph nodes on the right and two lymph nodes on the left). The cervix, fallopian tubes, and ovaries were macroscopically normal. Histologically, the tumor consisted of well-differentiated epidermoid cells with union bridges and dyskeratotic maturation invading the middle third of the myometrium. The diagnosis of a well-differentiated, invasive SCC of the endometrium was confirmed. There was no differentiation of adenocarcinoma, and no squamous metaplasia or dysplasia was observed. No tumor cells were seen in the cervix, the fallopian tubes, the ovaries, the omentum, or the lymph nodes.

The histoprognostic grade was classified as FIGO (International Federation of Gynecology and Obstetrics) stage IB, corresponding to pT1N0M0. The patient received three cycles of docetaxel (75 mg/m^2^, day 1, pump in) combined with carboplatin (200 mg/m^2^, day 1, drop in) chemotherapy.

After 3 and 6 months of follow-up, the patient was well, without recurrence.

## Discussion

We report a case of PSCC of the endometrium associated with HPV in a young Togolese woman. This is a very rare localization, and cases previously reported in the literature concerned postmenopausal women [5]. The young age of our patient makes this an exceptional case.

The frequency of PSCC of the endometrium is unknown, but Lee and Choi reported a frequency of PSCC of the endometrium of 0.7% of all endometrial tumors [5]. It is difficult to describe the pathogenesis of PSCC of the endometrium. There are several theories described in the literature, but none are formally accepted [6, 7]. In our patient, we did not observe squamous metaplasia or dysplasia, and the appearance of the cervix was normal. The presence of HPV in our patient might explain the pathogenesis of PSCC observed; however, we could not confirm this possibility. HPV infection plays an important role in the pathogenesis of squamous neoplasia of the cervix, but its role in PSCC of the endometrium remains controversial [2, 3]. It should be noted that Kataoka *et al*. demonstrated the presence of HPV type 31 by PCR in a patient with PSCC of the endometrium [3, 8]. However, there are no other reports showing a clear association between HPV and PSCC of the endometrium. Horn *et al*. performed HPV typing in eight patients with PSCC of the endometrium [9]. These authors noted that there was only one positive case with HPV type 16, but they could not establish the involvement of HPV type 16 in the genesis of this cancer [9, 10]. We therefore summarize that HPV may not be the main causative factor in PSCC of the endometrium. In our patient, Ki-67 labeling was high. The detection of ER and PR as prognostic indicators of PSCC of the endometrium remains uncertain [11, 12].

Total hysterectomy with bilateral salpingo-ovariectomy is the first-line treatment of choice, especially for postmenopausal women [2, 4, 8]. The efficacy of radiotherapy and chemotherapy as adjuvant therapies remains highly controversial [12, 13]. Epidermoid carcinoma has a poor prognosis compared with adenocarcinoma of the endometrium [12]. The 5-year survival rates for stages I, II, III, and IV of endometrial adenocarcinoma are 89.1%, 78.8%, 57.8%, and 22.8%, respectively [14]. However, the survival rates for squamous cell carcinoma of the endometrium are difficult to assess because of its scarce occurrence [1, 3]. With a survival period of 14 to 36 months, the 1-year survival rates for primary stages I, III, and IV of endometrial carcinoma are 80%, 20%, and 0%, respectively [7, 14]. PSCC of the endometrium has been reported to have a poorer prognosis than endometrioid carcinoma [4, 14]. The prognosis depends mainly on the tumor stage. Our patient was in FIGO stage IB, and she underwent a hysterectomy with adjuvant chemotherapy and had radiotherapy performed in a neighboring country (Ghana). Togo does not have a radiotherapy unit, and patients who require therapy are often sent to Ghana. It should also be noted that not all patients referred for radiotherapy can afford therapy, owing to the cost. There is no health insurance for anyone in Togo, and most of the population is of modest socioeconomic status. At a 3-month follow-up, the patient was in good health, without recurrence or metastasis.

## Conclusion

Pure primary endometrial squamous cell carcinoma is an extremely rare malignancy of the corpus uteri. The pathogenesis of this endometrial cancer is complex, and its association with HPV does not explain its genesis. Diagnosis of this rare entity is based on careful pathologic review of the hysterectomy specimen. The underlying etiology or inciting factors leading to this condition have yet to be determined. More studies are needed to address the concern about the extension of primary surgical treatment and the efficacy of adjuvant therapy in this disease.

## Abbreviations

FIGO: International Federation of Gynecology and Obstetrics
HE: Hematoxylin and eosin
HPV: Human papillomavirus
IHC: Immunohistochemistry
Ki-67: Antigen of cell multiplication
KL1: Anti-pancytokeratin 1
PR: Progesterone receptor
PSCC: Primary squamous cell carcinoma
SCC: Squamous cell carcinoma
